# The complete chloroplast genome of paradox (*Juglans major × J. regia*), an interspecific hybrid in China

**DOI:** 10.1080/23802359.2020.1765212

**Published:** 2020-05-14

**Authors:** Xiao-Bo Song, Qing-Guo Ma, Ye Zhou, Ying-Ying Chang, Jun-Pei Zhang, Dong Pei

**Affiliations:** State Key Laboratory of Tree Genetics and Breeding, Key Laboratory of Tree Breeding and Cultivation of National Forestry and Grassland Administration, Research Institute of Forestry, Chinese Academy of Forestry, Beijing, China

**Keywords:** Complete chloroplast genome, *Juglans*, phylogenetic analysis

## Abstract

Paradox is one of the most important rootstock resources in USA and China walnut industry. In this study, we characterized the complete chloroplast genome of Paradox (*Juglans major* × *J. regia*) based on next generation sequencing. The circular complete chloroplast genome was 160,324 bp in length, containing a large single-copy (LSC) region of 89,852 bp, and a small single-copy (SSC) region of 18,410 bp. These two regions were separated by a pair of inverted repeat regions (IRa and IRb) of 26,031 bp each. A total of 131 functional genes were encoded, consisted of 87 protein-coding genes, 36 tRNA genes, and 8 rRNA genes. The overall GC content of the chloroplast genome was 36.1% and the GC contents of the LSC, SSC, and IR regions were 33.7, 29.9, and 42.6%, respectively. The phylogenetic analysis by Neighbor-Joining showed that Paradox was relatively closely related to *J*. *major* compared to other species of *Juglans* genus. This complete chloroplast genome will provide valuable insight into evolution, molecular breeding, and phylogenetic analysis of *Juglans* species.

*Juglans* (Juglandaceae) is a genus of 20 species of trees, several species of which has been introduced around the world where it is used as food, timber and traditional medicines (Kim et al. 2006; Mabberley 2008). Paradox was developed through artificial hybridization between California walnut (*Juglans hindsii*) and Persian walnut (*J. regia*). Now, the name is commonly applied to any black walnut – Persian walnut hybrid (between sect. Rhysocaryon and sect. Juglans) (Potter et al. 2002). Paradox has become one of the most important rootstock resources in USA and China walnut industry (Suo et al. 2012). Although complete chloroplast genome sequencing of several species from *Juglans* genus have been completed (Hu et al. 2016, 2017), few genetic and genomic studies were carried out on interspecific hybrids. Therefore, we reported the complete chloroplast genome of Paradox (*J. major* × *J.regia*) based on Illumina sequencing data, which would be helpful for its evolution, molecular breeding, and phylogenetic analysis of this species.

Leaves of an Paradox individual were collected from Luoyang city, Henan Province (34°27′45″N；113°40′48″E) and stored at State Key Laboratory of Tree Genetics and Breeding with specimen code of Znq-1. A shotgun library was prepared and sequenced on the Illumina Hiseq X Ten Sequencing System. In total, 4.8 G raw reads were obtained, quality-trimmed and assembled for the chloroplast genome using the program ABySS v2.1.5 (Jackman et al. 2017), with *J. regia* (GenBank: MF167464) (Dong et al. 2017) as the initial reference. The genome was annotated using plann 1.1.2 (Huang and Cronk 2015) and confirmed with Apollo v2.3.1 (Lee et al. 2009). The finally annotated chloroplast genome has been deposited into GenBank with the accession number MT246265.

The complete chloroplast genome of *J. major* × *J. regia* is 160,324 bp in length and is comprised of a pair of inverted repeat (IR) regions (26,031 bp each), a large single-copy (LSC) region of 89,852 bp, and a small single-copy (SSC) region of 18,410 bp. The chloroplast genome encoded a total of 131 genes, including 87 protein-coding genes, 36 transfer RNA genes (tRNA), and 8 ribosomal RNA genes (rRNA).

A phylogenetic analysis was performed based on 11 complete chloroplast genomes of Juglandaceae (*J. major*, *J. cinerea*, *J. nigra*, *J. hindsii*, *J. regia*, *J. sigillata*, *J. cathayensis*, *J. mandshurica*, *J. hopeiensis*, *Annamocarya sinensis,* and *Cyclocarya paliurus*) and 1 species from Betulaceae (*Corylus chinensis*) as outgroups. All of the cp genome sequences were obtained from NCBI and aligned by using MAFFT v7.313 (Katoh and Standley 2013). The Neighbor-Joining (NJ) tree was constructed with RAxML v8.2.11 (Stamatakis 2014) and the branch support was estimated with 1000 bootstrap replications. The result indicated that Paradox was found to be relatively closely related to *J. major* compared to other species of *Juglans* genus (Figure 1).

**Figure 1.**
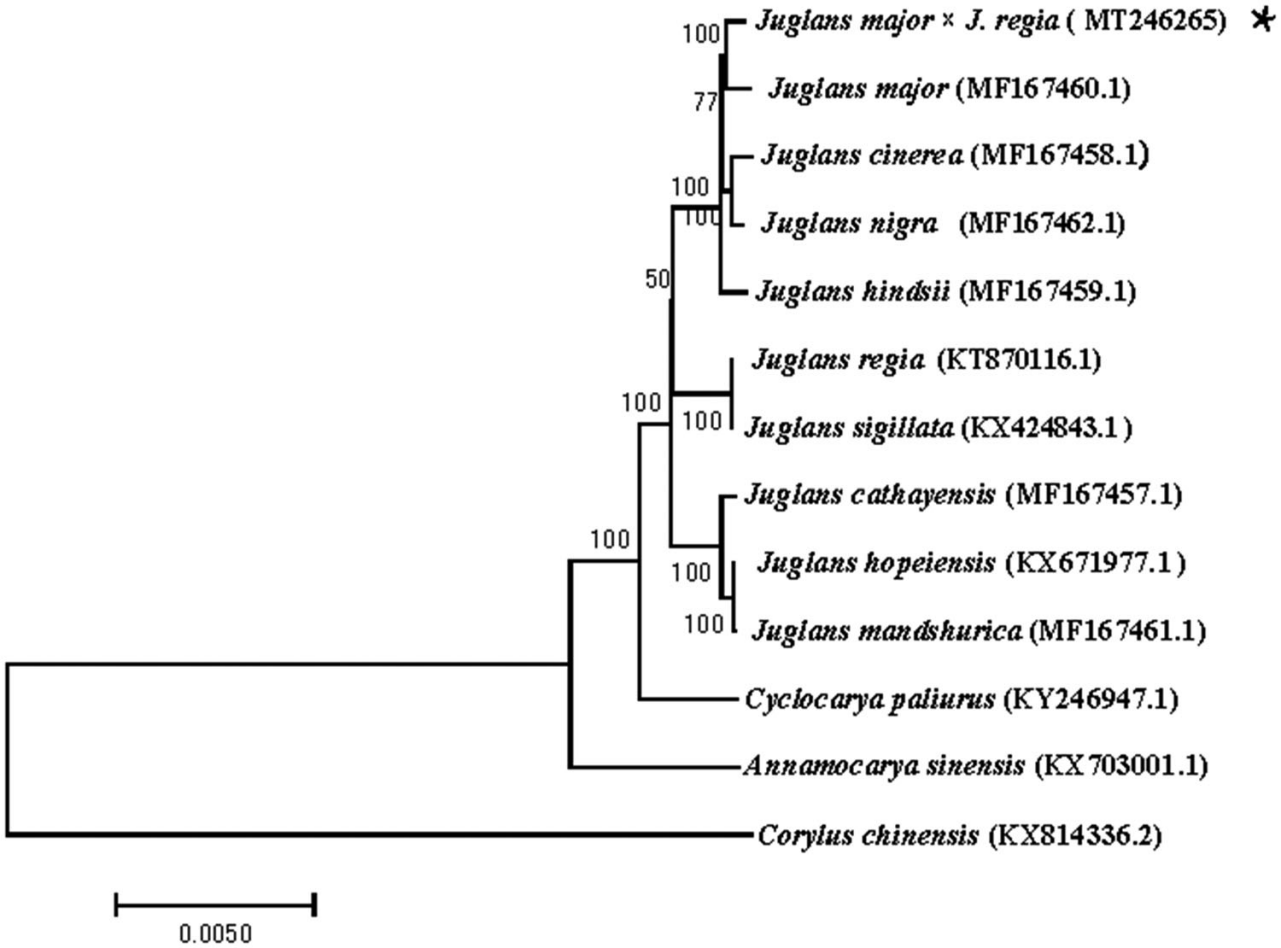
Neighbor-joining (NJ) tree based on the complete chloroplast genome sequences of 13 species. The numbers on the branches are bootstrap values.

## Data Availability

The data that support the findings of this study are openly available in GenBank at [https://www.ncbi.nlm.nih.gov/genbank/], reference number [MT246265].
